# Management of high-energy tibial shaft fractures using the hexapod circular external fixator

**DOI:** 10.1186/s12893-021-01106-5

**Published:** 2021-02-21

**Authors:** Yanshi Liu, Jialin Liu, Maimaiaili Yushan, Zhenhui Liu, Tao Zhang, He Ma, Chuang Ma, Aihemaitijiang Yusufu

**Affiliations:** 1 Department of Microrepair and Reconstruction, The First Affiliated Hospital of Xinjiang Medical University, Urumqi, Xinjiang China; 2 Department of Prosthodontics, The First Affiliated Hospital of Xinjiang Medical University, Urumqi, Xinjiang China; 3 Department of Orthopaedics and Trauma, Tianjin Hospital, Tianjin, China

**Keywords:** External fixation, Hexapod external fixator, Tibial shaft fractures, Trauma-control

## Abstract

**Background:**

The hexapod external fixator (HEF) is increasingly used for high-energy tibial shaft fracture care as more general orthopedic surgeons are gaining expertise of this versatile device. The purpose of this study was to evaluate the clinical effectiveness of the HEF for definitive management in patients with high-energy tibial shaft fractures.

**Methods:**

The study was conducted on 34 patients with tibial shaft fractures who were admitted or referred to our institution and consented to HEF treatment from Jan 2016 to June 2019, including 27 males and 7 females with a mean age of 39 years (range 18 to 65 years). Patients' clinical and radiological data, and the final clinical outcomes at a minimum of 12 months follow-up were collected and retrospectively analyzed. All complications were documented according to Paley’s classification. The clinical outcomes were evaluated using the Association for the Study and Application of the Method of Ilizarov criteria (ASAMI) at the last clinical visit.

**Results:**

All patients remained in the HEF for a mean of 26 weeks (range 15 to 52 weeks) and acquired complete bone union. The satisfactory alignment was achieved in all patients, and all the patients were able to perform daily activities with no difficulty at the last clinical visit. Complications included pin tract infection (44%), delayed union (6%), nonunion (3%), and joint stiffness (3%). The ASAMI bony result was excellent in 31 patients and good in 3. The ASAMI functional result was excellent in 27 patients, good in 6, and fair in 1.

**Conclusions:**

Definitive management using the hexapod external fixator is an alternative and effective method for high-energy tibial shaft fractures, including technical advantages of early trauma-control, the versatility of achieving excellent alignment, and the continuity of device until bone union.

## Background

The tibial shaft fractures are often the result of high-energy trauma, and most cases suffered significant soft tissue damage [1, 2]. The optimal definitive management remains controversial and a challenging orthopedic problem in the clinical scenario. Traditional treatments of these severe injuries involved plaster splint, plates and screws, intramedullary nail, external fixation, or a combination of these techniques depending on the fracture specifics [3, 4].

In most cases, intramedullary nail fixation acts as the gold standard, while external fixation plays a safe and effective role where internal fixation is impossible or inadvisable [5]. Many authors accept that stability is paramount for circular fixators to high energy injuries management, but a smaller physical interference of the device is preferred. The hexapod external fixator (HEF), such as the Taylor spatial frame (TSF), is a symmetric configuration of the Stewart platform that consists of 2 rings or partial rings connected by 6 telescopic struts at special universal joints [6, 7]. The HEF provides all the advantages of multiplanar fixation of the Ilizarov system and is equipped with the versatility of spatial deformities correction without the alteration of frame construct. The HEF was initially developed to correct complex multi-planar deformities, and its use subsequently expanded to treat the bone nonunion and fractures [8–12]. As for complex low limb trauma, especially in cases with poor surrounding soft tissues and significant extremity deformities, the HEF had become an attractive option in recent years.

Although lots of studies have shown excellent deformity correction of the HEF [10–15], there is little published data demonstrating the clinical outcomes of fractures [8, 9, 16]. As more general orthopedic surgeons are becoming familiar with this versatile device, the HEF is gaining popularity in high-energy tibial shaft fractures care. The purpose of this study was to evaluate the clinical effectiveness of the HEF for definitive management in patients with high-energy tibial shaft fractures.

## Methods

Thirty-four patients with high-energy tibial shaft fractures, who were admitted or referred to our institution and consented to hexapod external fixator (Tianjin Xinzhong Medical Instrument Co., Ltd., Tianjin, China) treatment from Jan 2016 to June 2019, were included in this retrospective study. Inclusion criteria were open fractures, closed fractures with poor surrounding soft tissues, or polytrauma with an Injury Severity Score (ISS) ≥ 16. Patients with pathological fractures, any other illness that can affect bone healing (such as diabetes, osteoporosis, etc.), age > 65, poor compliance, and patients treated initially with the HEF but converted to internal fixation were excluded.

The HEF treatment was based on a thorough discussion in our medical team for the best potential outcomes. Indications of the application of HEF included high-energy complex fractures, fractures with severe soft-tissue damage that was not suitable for internal fixation and followed by gradual deformity correction, and fractures that needed delayed soft tissue reconstruction.

The present study contains 27 males and 7 females with a mean age of 39 years (range 18 to 65 years). Informed consent was obtained from all patients for their data to be documented and published in our study. This study was approved by the Ethical Committee of our institution.

The injury mechanism was road traffic accident in 26 patients, fall from height in 4 patients, sports injury in 2 patients, and crushing injury caused by a heavy object in 2 patients. The fractures were subdivided depending on the OTA classification system. The 27 open fractures were classified using the Gustilo and Anderson classification [17]. In those closed fractures, 1 patient suffered compartment syndrome, 3 patients showed hemorrhagic fracture blisters on presentation to our institution, 2 patients failed closed reduction with plaster immobilization, and postoperative malalignment was observed in the other one patient treated with the monolateral fixator elsewhere (Table 1).

**Table 1:** Summary of all patients with tibial shaft fractures treated with TSF

Number of patients	34
Mean age (years)	39 (18–65)
Sex	
Male	27 (79%)
Female	7 (21%)
Injury mechanism	
Road traffic accident	26 (76%)
Fall from height	4 (12%)
Sports injury	2 (6%)
Crushing injury caused by heavy object	2 (6%)
Injury Severity Score (ISS)	19 (16–30)
Open/closed fracture	
Open	27 (79%)
Closed	7 (21%)
Associated injury	
Ipsilateral humeral fracture	2 (6%)
Ipsilateral femoral fracture	2 (6%)
I psilateral ankle fracture	2 (6%)
Ipsilateral calcaneal fracture	1 (3%)
Ipsilateral metatarsal fracture	1 (3%)
Contralateral tibial fracture	1 (3%)

Nine patients had associated injuries, including an ipsilateral humeral fracture in two patients, ipsilateral femoral fracture in two patients, ipsilateral ankle fracture in two patients, ipsilateral calcaneal fracture in one patient, ipsilateral multiple metatarsal fractures in one patient, and contralateral tibial fracture in one patient (Table 1).

Postoperative data were recorded, including surgical procedure time, time to the satisfactory reduction achieved, external fixation time, complications, duration of follow-up, and outcomes at the last follow-up. All preoperative and postoperative radiographs in both the frontal and sagittal planes were used to assess the fracture type, deformities, final alignment, and union. All patients were followed up at least 12 months after removing the TSF.

Difficulties that occur intraoperatively and during postoperative treatment were subclassified according to Paley [18]. Pin site infections were classified into 6 grades (grade 0 to grade V) [19]. The clinical results were assessed by the Association for the Study and Application of the Method of Ilizarov criteria (ASAMI) at the last clinical visit [20].

### Surgical technique

All the surgical procedures were conducted by the same team, and all the patients acquired preventative cephalosporin antibiotics perioperatively. Radical debridement and irrigation were performed before the installation of the HEF in the open fractures. Four patients with Type I wound and 9 patients with Type II wound were closed primarily within 8 h, while the other 3 Type II wound had delayed primary closure. Eight patients with Type IIIA wound had split-thickness skin grafting, and the other 3 with Type IIIB wound had a rotational flap. The patient with closed fracture suffered compartment syndrome underwent fasciotomy followed by skin grafting. The other patients who failed closed reduction underwent the definitive management of the TSF. The associated fractures in 9 patients were managed simultaneously or another subsequent surgical procedure considering the specifics. According to the treating surgeon’s discretion, all the procedures were performed between 1 and 12 days after injury.

HEF was applied under the image intensifier control. The diameter of the two rings was determined by the injured extremity, ensuring that there is enough space for additional local care. The “ring-first” technique was used in all cases. The rings were attached to each bony segment and were perpendicular to the long axis of the corresponding bony segment in an orthogonal manner. Two or three transverse 1.8-mm smooth transosseous wires and one or two 6-mm half pins were inserted in each bony fragment. The wires were tensioned to 110 kg. The two rings were mounted to these wires and half pins on each side independently, and then the six struts were attached to the nonparallel rings. The fracture was reduced manually to an acceptable position, and the struts were locked subsequently. Struts equipped with a fast closure mechanism were preferred due to the ease of manipulation.

### Postoperative management

Standard postoperative orthogonal anteroposterior (AP) and lateral radiographs were used to evaluate the residual deformities. Thirteen parameters needed by the computer system were calculated based on these X-rays. The total residual program of the HEF was performed in all cases. All the residual deformities were corrected by gradual strut adjustment postoperatively, according to the electronic prescription.

Isometric muscle and joint range of motion (ROM) exercise within the tolerance of pain were encourage for all patients on the second day after surgery, and a rigid shoe with an elastic band was used to keep the foot in a neutral position to prevent ankle equines contractures. Daily pin site care with alcoholic chlorhexidine were recommended to avoid pin tract infection. The other degrees of wire and pin tract infection were classified and managed according to the Dahl classification [19].

The patients were followed up weekly until total corrections were achieved and then monthly. The HEF was removed when sufficient union (corticalization in 3 of 4 cortices) was demonstrated in radiographs and a clinical assessment was completed by the treating surgeon. All patients were requested to put on the functional brace for four weeks to prevent refracture.

### Statistical analysis

Statistical analysis was performed with the SPSS 22.0 (IBM Corp, USA) software. Continuous variables were analyzed by Independent-samples T-tests and expressed as the mean, standard deviation (SD), and range of the observations. And the count variables were analyzed by the Chi-square or Fisher’s test, expressing as number. Statistically significant difference was set at P < 0.05.

## Results

The fracture classifications are shown in Tables 2 and 3. The road traffic accident was the most common mechanism of injury (26 cases, 76%). The mean ISS was 19 ± 4.1 (range 16 to 30) (Table 1).

**Table 2:** OTA classifications of fractures in this study

Fracture type			Number	Open
4 (Tibia)	2 (Diaphyseal)	*A (simple)*		
		-A1	2	2
		-A2	2	1
		-A3	1	1
		*B (some comminution)*		
		-B1	8	7
		-B2	7	6
		-B3	2	2
		*C (highly comminuted/segmental)*		
		-C1	5	3
		-C2	3	2
		-C3	4	3

**Table 3:** Gustilo and Anderson classifications of the 27 open fractures in this study

Classifications	Number	Percentage (%)
Type I	4	15%
Type II	12	44%
Type IIIA	8	30%
Type IIIB	3	11%
Type IIIC	0	0

Damage-control was initially managed by a monolateral external fixator in 18 cases (53%), and the HEF was initially applied to manage 4 patients (12%). Nine fractures (26%) were treated by a skeletal traction pin for initial stabilization at hospital admission. The time elapsed since the primary care to HEF applied presented at an average of 4 days (range 1 to 12 days). The physiological instability and associated soft-tissue injury contributed to the delayed application of the HEF.

Patients underwent a mean surgical procedure time of 113 min (range 80 to 150 min), and the mean time to reduction was 8 days (range 5 to 14 days). A satisfactory alignment was achieved in all patients. The mean preoperative translation and angulation were 16.4 mm (range 5 to 38 mm) and 5.5° (range 0 to 12°) on the AP radiograph. The mean preoperative translation and angulation were 10.8 mm (range 0 to 26 mm) and 4.3° (range 0 to 13°) on the lateral radiograph. The mean preoperative leg length discrepancy was 8.2 mm (range 0 to 19 mm). The mean final translation and angulation were 0.6 mm (range 0 to 5 mm) and 0.5° (range 0 to 2°) on the AP radiograph after correction. The mean final translation and angulation were 0.5 mm (range 0 to 4 mm) and 0.4° (range 0 to 3°) on the lateral radiograph after correction. Although a mean of 0.9 mm (range 0 to 5 mm) leg length difference was showed in the postoperative radiographs, there was no clinical leg length discrepancy. There were statistically significant differences between the preoperative deformities and postoperative correction achieved (p < 0.05) (Table 4).

**Table 4:** Comparison of preoperative deformities and postoperative correction achieved

Residual deformities	PD	PCA	t	P-value
Residual translation on AP view (mm)	16.4 (5–38)	0.6 (0–5)	10.139	P < 0.001
Residual angulation on AP view (°)	5.5 (0–12)	0.5 (0–2)	8.978	P < 0.001
Residual translation on lateral view (mm)	10.8 (0–26)	0.5 (0–4)	9.395	P < 0.001
Residual angulation on lateral view (°)	4.3 (0–13)	0.4 (0–3)	8.985	P < 0.001
Residual leg length discrepancy (mm)	8.2 (0–19)	0.9 (0–5)	9.078	P < 0.001

*PD* preoperative deformities, *PCA* postoperative correction achieved

**Fig. 1:**
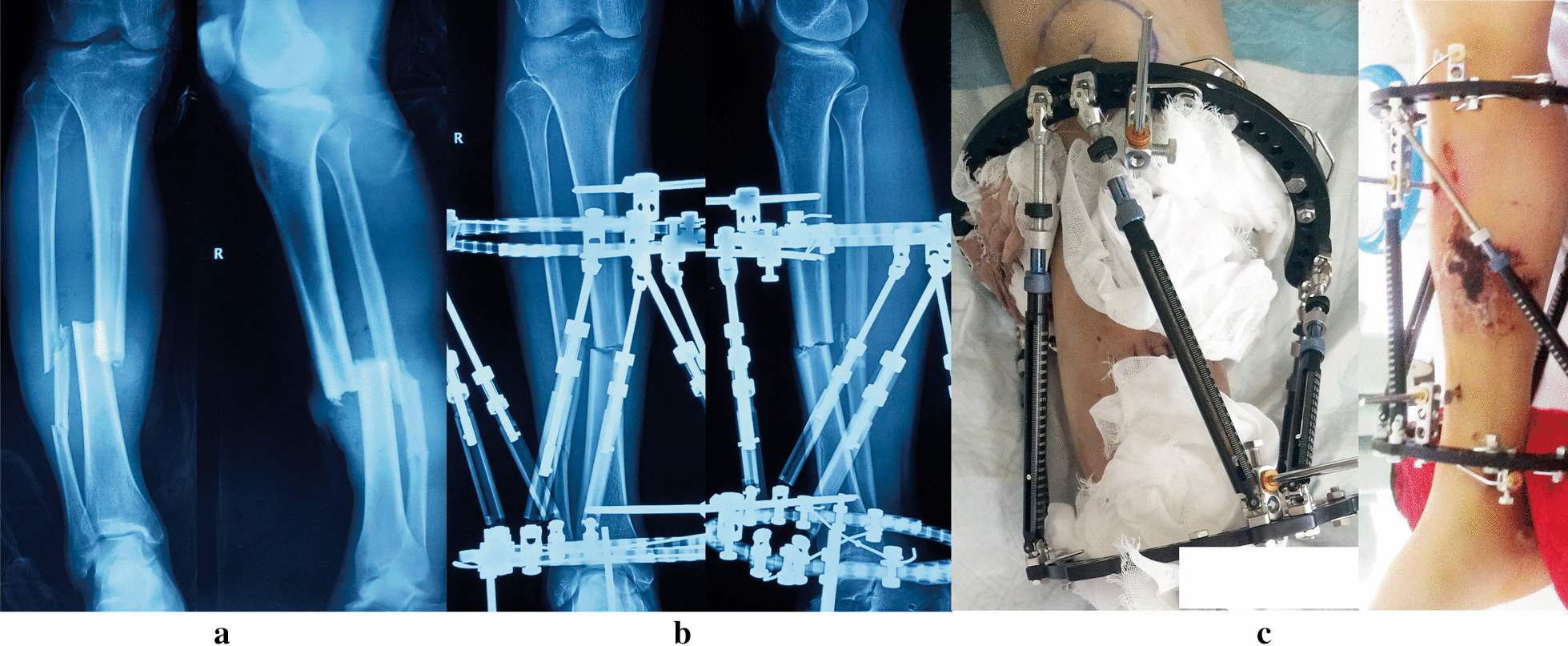
Images of a 54-year-old man with posttraumatic multidimensional deformity in tibia and fibula treated by the TSF. **a** Posttraumatic AP and lateral views of X-rays. **b** Radiographs immediately after application of TSF. **c** Patient in TSF with preoperative hemorrhagic fracture blisters seen

**Fig. 2:**
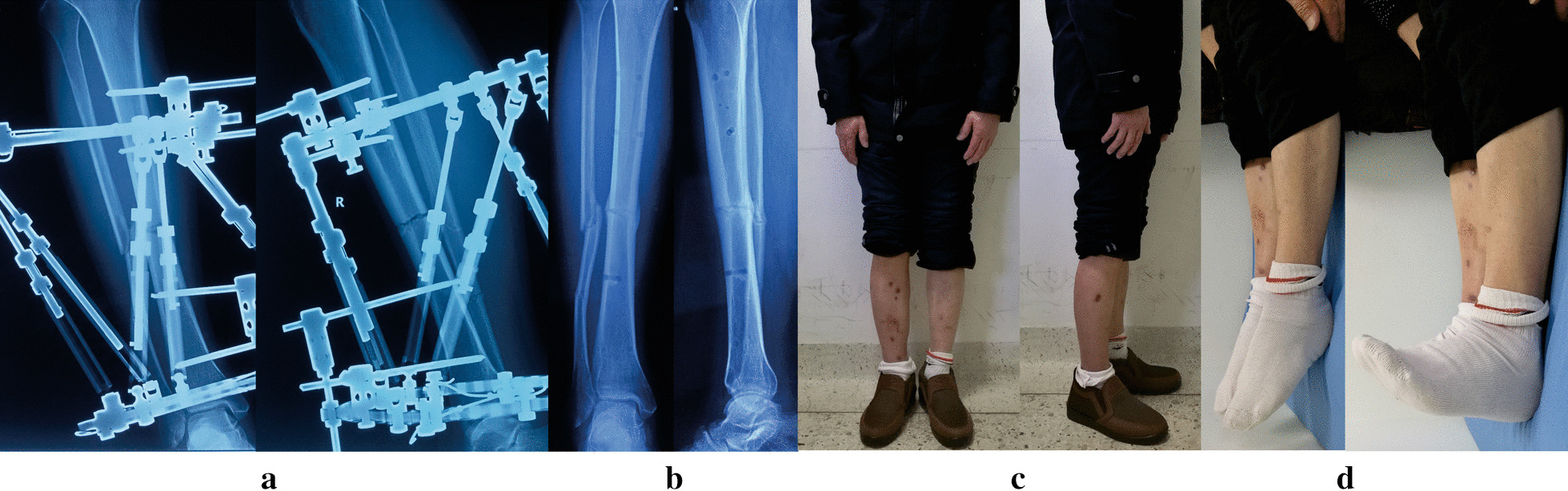
Images of the same patient shown in Fig. 1. **a** Radiographs after final correction. **b** Radiographs one months later after removal of TSF. **c**, **d** Clinical follow-up images, obtained at 12 months after TSF removal

**Fig. 3:**
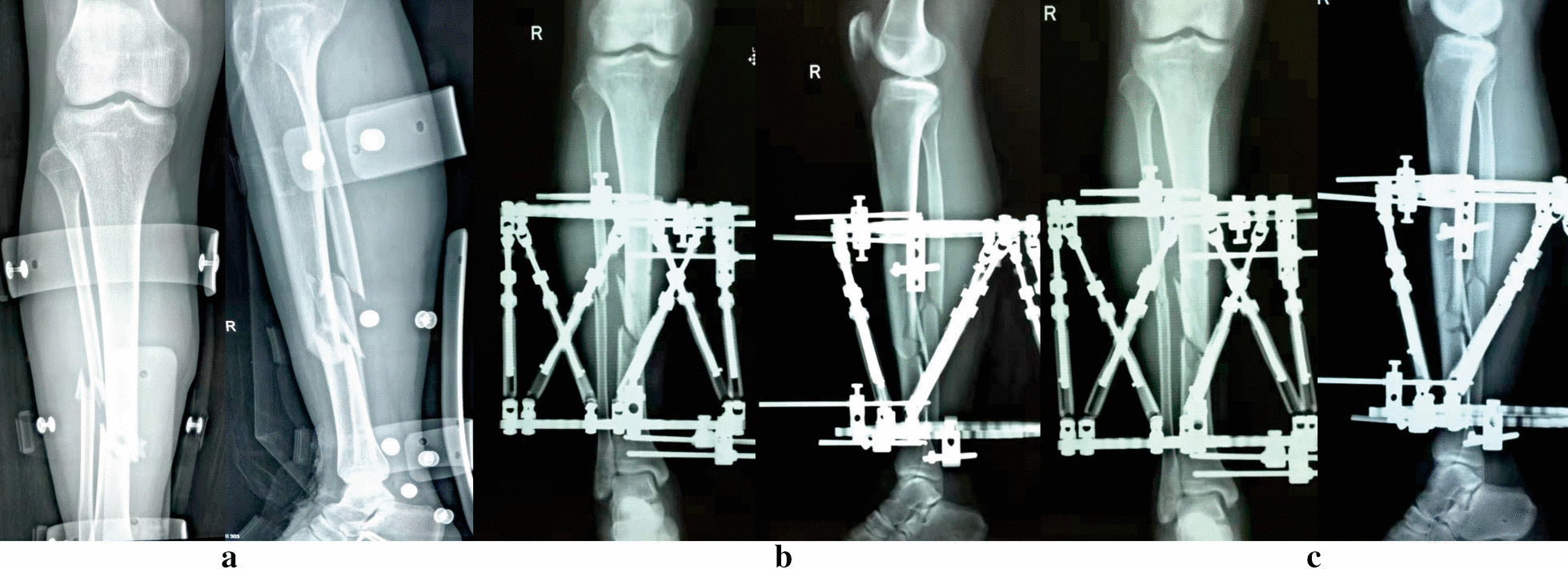
Images of a 32-year-old man suffered posttraumatic open tibial and fibular fractures treated by the TSF. **a** Posttraumatic AP and lateral views of X-rays. **b** Radiographs immediately after application of TSF. **c** Radiographs after final correction

**Fig. 4:**
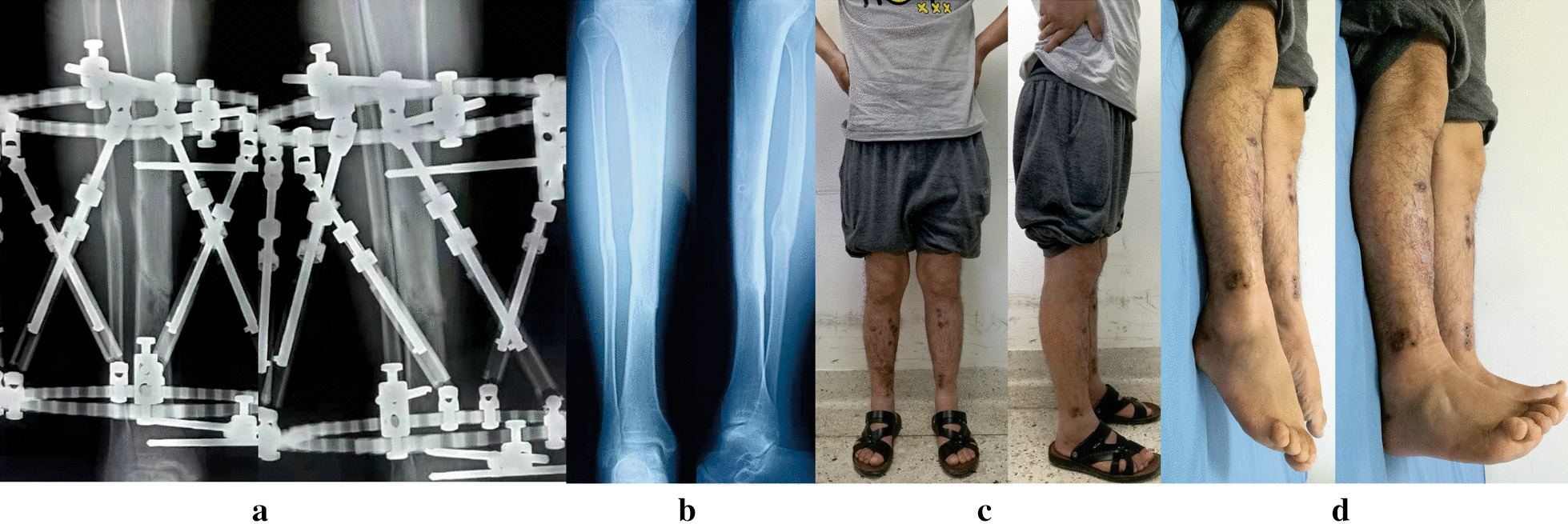
Images of the same patient shown in Fig. 3. **a** Radiographs after 6 months from TSF application, revealing hone healed. **b** Radiographs 6 months later after removal of TSF. **c**, **d** Clinical follow-up images, obtained at 13 months after TSF removal

All patients (100%) achieved bone union with a mean time of 26 weeks (range 15 to 52 weeks). Primary fracture union was observed in 28 patients (82%) with a mean time of 24 weeks (range 15 to 32 weeks). Three patients (9%) suffered bone loss due to debridement and were successfully managed by acute shortening and relengthening, the mean time to healing was 31 weeks (range 28 to 35 weeks). In these 3 cases, bone lengthening and anatomic alignment were achieved by the HEF strut adjustments. The other 3 patients (9%) required further intervention procedures and united at a mean of 43 weeks (range 37 to 52 weeks). All patients were followed for an average of 15 weeks (range 12 to 26 weeks) after removing the TSF. (Figs. 1, 2, 3, 4).

There were no intraoperative complications. The most common postoperative complication was pin tract infection (44%) as expected in this study. 13 patients suffered superficial pin tract infection and treated by daily pin site care and oral antibiotics, 2 patients suffered deep pin tract infection and successfully solved by pin replacement and intravenous antibiotics, and no patient developed sequestrum requiring debridement (Table 5).

**Table 5:** Summary of complications

Complications	Number	Percentage (%)
Pin tract infection	15	44%
Joint stiffness	1	3%
Delayed union	2	6%
nonunion	1	3%
Total	19	
Total patients affected	12	
Complication rate	35.3%	

Two cases (6%) suffered delayed union, which was successfully treated by the “accordion maneuver” technique. The frames were thereby applied for 37 weeks and 40 weeks, respectively. One patient (3%) with a severely comminuted fracture developed to nonunion and finally achieved bone union by autogenous iliac crest bone grafting; the TSF was used continuously for 52 weeks. One patient (3%) suffered ankle joint stiffness after removing the TSF and successfully managed by a surgical release. No patients developed a loss of reduction, a malunion, and a neurovascular injury (Table 5).

All the patients were able to perform daily activities without significant difficulties when last seen. Thirty-two patients were not experiencing any pain, but the other 2 patients felt mild pain sometimes at the rotational flap site without any analgesics requirement. According to the ASAMI scoring, the bony result was excellent in 31 patients (91.18%), good in 3(8.82%). The functional result was excellent in 27 patients (79.41%), good in 6 (17.65%), fair in 1 (2.94%) (Table 6).

**Table 6:** Results of ASAMI scores

		Excellent	Good	Fair	Poor	Failure
ASAMI	Bone grade	31	3	0	0	–
	Function grade	27	6	1	0	0
ASAMI criteria						
*Bone results*						
Excellent: Union, no infection, deformity < 7°, limb length discrepancy (LLD) < 2.5 cm						
Good: Union plus any two of the following: absence of infection, deformity < 7°, LLD < 2.5 cm						
Fair: Union plus any one of the following: absence of infection, deformity < 7°, LLD < 2.5 cm						
Poor: Nonunion/refracture/union plus infection plus deformity > 7° plus LLD > 2.5 cm						
*Functional results*						
Excellent: Active, no limp, minimum stiffness (loss of < 15°knee extension/ < 15°ankle dorsiflexion) no reflex sympathetic dystrophy (RSD), insignificant pain						
Good: Active, with one or two of the following: limb, stiffness, RSD, significant pain						
Fair: Active, with three or all of the following: limb, stiffness, RSD, significant pain						
Poor: Inactive (unemployment or inability to return to daily activities because of injury)						
Failure: Amputation						

## Discussion

The optimal treatment for high-energy tibial fractures remains a controversial issue. High-energy tibial fractures associated with poor surrounding soft tissues pose potential threats to bone healing and a high risk of infection. Multilevel stabilization and minimal soft tissue disruption should be comprehensively considered when treating these fractures to maintain the biological environment. The definitive fixation modality is informed by the fracture pattern, skin condition, and the skill set and preferences of the treating surgeon.

Although internal fixation contributes to anatomic reduction, it is the most invasive form with potential infection risk. External fixation, which can preserve the biomechanical microenvironment of fracture healing, plays an important role in managing unstable fractures, especially for high-energy injuries with poor soft tissue [5, 21–23]. The previous study has demonstrated that the clinical results of tibia fractures treated by definitive circular fixation are better than by monolateral or hybrid external fixation, and there is a comparable effect compared with intramedullary nailing [24]. Although no evidence determines the superior circular fixator form, the hexapod circular fixation has become an attractive option for trauma-control and definitive management due to its versatility of multiplanar deformities correction without changing the frame [8–12].

In the present study, the author used the HEF to definitively treated a group of high-energy tibial shaft fractures. This method allows immediate trauma-control of the fracture with acute or gradual reduction, facilitates access to soft tissues for reconstruction, and represents the ultimate form of indirect reduction techniques. Gordon et al. [25] reported that a monolateral device for managing tibial shaft fractures poses a statistically significant risk of loss reduction. In this study, satisfactory alignment was achieved in all patients, and no loss of reduction was observed due to the higher stability of the HEF. Besides, the HEF can be rapidly applied in experienced hands without worrying about fracture reduction accuracy due to the ability of postoperative correction.

Our mean residual translation and angulation was 0.6 mm (0–5 mm) and 0.5° (0–2°) on the AP radiograph after correction, 0.5 mm (0–4 mm) and 0.4° (0–3°) on the lateral radiograph. Additionally, the residual leg length discrepancy was 0.9 mm (0–5 mm), and no significant leg length discrepancy was detected clinically. The little residual deformity showed in the present study, reflecting the versatility of the TSF for any postoperative deformities and providing the ability to achieve excellent alignment postoperatively.

Lots of published data showed significant complications such as pin tract infection, delayed union, nonunion, refracture, axial deviation, and joint stiffness [2, 5, 7, 23, 26–28]. In this study, as expected, the most common complication was pin tract infection. 15 patients (44%) had a pin tract infection and successfully managed by antibiotics or pin replacement. Patients with severe soft tissue swelling were the main cases, which may explain the increased infection rate. We speculate that the timing of surgery is the utmost factor, which was said to affect the risk of soft tissue related infections. The morbidity of pin track infection in our study (44%) matched that in the literature of Antoci et al. [29] (33%), Francesco et al. [30] (35%), and lower than Al-sayyad (63%) [31], Schalamon (52%) [32]. No patient developed sequestrum or osteomyelitis requiring debridement. Joint stiffness was observed in one patient, and a poor rehabilitation might account for it. There was no loss of reduction, malunion, and neurovascular injury.

Long bone fractures requiring stabilization with a circular fixator are relatively uncommon. Menakaya et al. [33] conducted a 5-year consecutive series study and concluded that TSF could act an essential role in complex tibial fractures. Sala et al. [34] used the TSF as a definitive fixation to treat 12 patients with shaft and distal femoral fracture, achieving good clinical and radiograph outcomes finally. Maarten et al. [35] definitively treated 102 diaphyseal fractures with hexapod circular external fixation, acquiring bone union in 101 cases with an average time of 25.6 weeks. In the present study, all patients achieved bone union, therein two delayed union was successfully managed by the “accordion maneuver” technique, and one nonunion solved by autogenous iliac crest bone grafting finally. Almost all patients were able to perform daily activities without significant difficulty. The patients in our study had an average external fixation time of 26 weeks, which matches the outcomes of Francesco et al. [7], Al-sayyad [31], and Potgieter et al. [35]. We, therefore, acknowledge that the functional and bony outcomes of frame fixation are good, especially for the complex injury. Our study of 34 patients, including 27 open fractures, compensating the existing literature about the hexapod external fixator for the care of high-energy long bone shaft fractures reporting good results.

As delineated above, according to our experience, early functional weight-bearing and active motion of adjacent joints are permitted when the HEF is used. With the abilities of deformity correction and definitive fixation simultaneously using the HEF, there is no need to expose the bone and thereby avoid the disadvantages of open surgery and contributing to satisfactory outcomes. The HEF provides not only the postoperative adjustment benefits to achieve excellent alignment before bone consolidation, but also all the advantages of external fixation. The hexapod configuration simplifies intraoperative fracture reduction, with which the surgeons can achieve perfect reductions without return to the operating room. The significant limitations of the HEF, we think, are the high economic burden and steep learning curve, especially for treating complex limb deformities. The treating surgeon must measure up to 13 parameters needed by the computer system, and even a minor error will affect the outcomes. To avoid the common complications of external fixation treatment such as pin site infection, meticulous attention and regular follow up care were recommended to ensure the desired outcomes.

The retrospective nature with a small sample size and the absence of a control group (such as the intramedullary nail for tibial shaft fracture care) that allows concluding the healing rates, complications, costs, and times are the two limitations of the present study. Despite these inherent limitations, this study reports a common group of patients with high-energy tibial shaft fractures that are recovering to pre-injury health status, promising a satisfactory outcome. Subsequent research is needed, in which the other promising alternative method is used for personalized management in each case and their results will be compared with the present control.

## Conclusion

Definitive management using the hexapod external fixator is an alternative and effective method for high-energy tibial shaft fractures, including technical advantages of early trauma-control, the versatility of achieving excellent alignment, and the continuity of device until bone union.

## Data Availability

The datasets analysed during the current study are available from the corresponding author on reasonable request.

## Abbreviations

HEF: Hexapod external fixator
TSF: Taylor spatial frame
ASAMI: Association for the Study and Application of the Method of Ilizarov criteria
ISS: Injury severity score
ROM: Range of motion
AP: Anteroposterior
